# An indicator approach to capture impacts of white-tailed deer and other ungulates in the presence of multiple associated stressors

**DOI:** 10.1093/aobpla/plx034

**Published:** 2017-07-22

**Authors:** Bernd Blossey, Andrea Dávalos, Victoria Nuzzo

**Affiliations:** 1 Department of Natural Resources, Fernow Hall, Cornell University, Ithaca, NY 14853, USA; 2 Natural Area Consultants, 1 West Hill School Road, Richford, NY 13835, USA

**Keywords:** Deer browse, indicator approach, invasive earthworms, multiple stressors, red oak, rodent

## Abstract

Management of ungulates is contested ground that lacks stakeholder agreement on desirable population sizes and management approaches. Unfortunately, we often miss information about extent of local impacts, for example on plant communities, to guide management decisions. Typical vegetation impact assessments like the woody browse index do not assess herbaceous plants, and differences in browse severity can be a function of deer density, deer legacy effects, localized deer feeding preferences and/or differences in plant community composition. Furthermore, in heavily affected areas, few remnant plants may remain for assessments. We used a sentinel approach to assess impact of white-tailed deer (*Odocoileus virginianus*), rodent attack, invasive earthworms and three invasive plants on survival and growth of 3-month-old red oak (*Quercus rubra*) individuals. We planted cohorts in 2010 and 2011 into deer accessible and fenced 30 × 30 m plots at 12 forests in New York State. We found year and site-specific effects with high deer herbivory of unprotected individuals (70–90 % of oaks browsed by deer versus none in fenced areas) far exceeding importance of rodent attacks. Oaks planted at low earthworm density sites were at significantly higher risk of being browsed compared with oaks at high earthworm density sites, but there was no detectable negative effect of invasive plants. Surviving oaks grew (~2 cm per year) under forest canopy cover, but only when fenced. We consider planting of oak or other woody or herbaceous sentinels to assess deer browse pressure a promising method to provide quantifiable evidence for deer impacts and to gauge success of different management techniques. The strength of this approach is that typical problems associated with multiple stressor impacts can be avoided, areas devoid of forest floor vegetation but under heavy deer browse pressure can still be assessed and the method can be implemented by non-specialists. Implementation of regular assessments can guide ungulate management based on meaningful evidence.

## Teaser

We developed a standardized metric to measure impacts of high white-tailed deer populations using red oak (Quercus rubra) seedlings. Our “sentinel approach” showed that deer browse reduced oak seedling survival and growth far more than invasive plants or invasive earthworms. Our approach can be used in areas devoid of vegetation but under heavy deer browse pressure, and the method can be implemented by non-specialists.

## Introduction

Many populations of mammals, including large predators and ungulates, are rapidly declining, often in response to shrinking habitats and excessive hunting pressure (Donlan *et al.* 2006; Maxwell *et al.* 2016). At the same time, a few adaptable ungulate species thrive in human-dominated landscapes in Europe, Australia, Japan, New Zealand and North America (Forsyth *et al.* 2010; Tanentzap *et al.* 2012; Foster *et al.* 2014; Howland *et al.* 2014; Perea *et al.* 2014; Iijima and Ueno 2016). These gains and losses of browsers and grazers, that function as important drivers of plant community composition, can have transformative consequences for ecosystems (Wardle and Bardgett 2004; Wardle *et al.* 2011). Management of species that thrive in human-dominated landscapes is contested ground (Frye 2006; Sterba 2012) and debates over the appropriate local or regional abundance of browsers and grazers are fraught with difficulties in balancing interests of different stakeholder groups.

An illuminating example of this problem is the success of white-tailed deer (*Odocoileus virginianus*) in North America. Recreational and commercial hunting brought the species to near extinction by the late 1800s until changes in hunting regulations allowed populations to rapidly recolonize their historic range (Leopold *et al.* 1947; Halls 1984). The return of white-tailed deer was welcomed by recreational hunters but as deer populations increased they quickly became local, and then regional, problems for forest regeneration, agriculture and conservation interests (Leopold *et al.* 1947; McCabe and McCabe 1984). The state wildlife agencies established to manage recovery of white-tailed deer were effective in rebuilding populations but philosophically and financially they continue to be poorly equipped to switch from conservation to management of damage and impact of abundant deer (Halls 1984; Brown *et al.* 2000; Rooney 2001; Jacobson *et al.* 2010).

Today there is widespread scientific consensus that white-tailed deer populations are too high over much of North America. The science documenting and forecasting the extensive and prolonged negative impacts of high deer browse pressure is clear and largely uncontested, and has been so for decades (Leopold *et al.* 1947; Côté *et al.* 2004; Binkley *et al.* 2006). Large deer populations not only affect crops and valuable timber species, but also create simplified and less diverse ecosystems with cascading negative impacts percolating through food webs affecting plants, insects, birds and ecosystem processes (Côté *et al.* 2004; Wardle and Bardgett 2004; McGraw and Furedi 2005; Martin *et al.* 2011; Nuttle *et al.* 2011; Schweitzer *et al.* 2014). Furthermore, deer appear to have an outsized influence on prevalence of tick-borne diseases, such as Lyme (Stafford *et al.* 2003; Raizman *et al.* 2013; Kilpatrick *et al.* 2014), and are implicated in facilitating spread of invasive plant species and invasive earthworms (Eschtruth and Battles 2009; Knight *et al.* 2009*b*; Kalisz *et al.* 2014; Dávalos *et al.* 2015*b*, *c*).

Despite the overwhelming scientific consensus on negative deer impacts, deer management agencies have not been able to devise effective approaches to reduce impacts. At the core of future deer impact management is the need to integrate scientific assessments and local documentation of the role of deer in shaping present day ecosystems, while recognizing that values of land and wildlife managers and of society are evolving (Jacobson *et al.* 2010). A review of published studies assessing deer ecosystem impacts in North America highlighted the absence of locally available evidence over much of North America (Russell *et al.* 2001). We have information about deer impacts in areas where experiments are being conducted, but methodological problems (short-term investigations, lack of replication, lack of repeat sampling of individuals, lack of incorporation of other stressors, etc.) plague many studies (Russell *et al.* 2001) and likely contribute to contentious debates. While new studies and reviews provide a more sophisticated and complete picture of deer impacts (McShea and Rappole 2000; Horsley *et al.* 2003; Côté *et al.* 2004; Allombert *et al.* 2005*a*, *b*; McGraw and Furedi 2005; Knight *et al.* 2009*a*; Heckel *et al.* 2010; Habeck and Schultz 2015) (see also Averill *et al.*, this issue), debates over population targets for deer herds continue unabated. Furthermore, the evidence available in scientific publications does not appear to percolate to deer hunters and decision makers (Flader 1974; Diefenbach *et al.* 1997; Frye 2006; Cambronne 2012; Sterba 2012).

An important impediment for state wildlife agencies to becoming accountable and for fulfilling their articulated holistic public trust obligations (Hare and Blossey 2014) and informing stakeholder debates is lack of locally available quantifiable evidence for the full extent of impacts associated with high deer populations. Scientific effort and studies are by design largely local, and questions are often raised whether results apply to other locales. Further complicating the issue is that (i) deer impacts are not necessarily a function of deer numbers, the metric often used to define landscape level population management goals (Putman *et al.* 2011), and more importantly (ii) deer are one of many stressors (Fisichelli *et al.* 2013) including pollution, plant and animal invasions, climate change and land-use history. Thus, it is difficult to determine whether high deer populations are the most important factor in explaining the demise of other biota leading to debates about appropriate management approaches.

Traditionally, a large number of methods and metrics have been proposed to assess deer impacts, including plant community composition (Habeck and Schultz 2015) (and see Averill *et al.* and Nuzzo *et al.*, this issue). Among the most well-known approaches are various iterations of the woody browse index originally developed for roe deer (*Capreolus capreolus*) management (Morellet *et al.* 2001; Pierson and DeCalesta 2015), and also combinations of woody browse and deer health indicators that should reflect habitat quality (Morellet *et al.* 2007). However, the woody browse index fails to measure impacts on herbaceous species that may be more sensitive to deer browse and are also unable to grow out of deer browse height. In North America, dozens of woody and palatable herbaceous species have been proposed as indicators of deer browse severity (Anderson 1994; Balgooyen and Waller 1995; Webster and Parker 2000; Williams *et al.* 2000; Fletcher *et al.* 2001; Webster *et al.* 2001; Kraft *et al.* 2004; Kirschbaum and Anacker 2005; Krueger and Peterson 2006; Pierson and DeCalesta 2015; Royo *et al.* 2016), but none is in regular use by wildlife managers to set deer population targets. Implementation of many of these assessment methods requires presence of specimens to measure height or flowering of individuals. Assessment of select species can provide useful information (see Nuzzo *et al.*, this issue) without requiring specialized knowledge that most individual landowners may not have (particularly for woody browse and plant community assessments). However, in some of the most heavily affected areas, or regenerating woodlands, depauperate plant communities may make finding sufficient individuals of species proposed as sensitive deer browse indicators largely impossible.

We were interested in developing an approach that would allow individual landowners, NGOs and other land managers to assess impacts of white-tailed deer on important species, and also decouple deer impacts from influence of other stressors (Fisichelli *et al.* 2013; Dávalos *et al.* 2015*a*, *b*). There is a long history in applied ecology for using indicator species (Dale and Beyeler 2001; Bachand *et al.* 2014) and increasingly indicator species analyses (see also Averill *et al.*, this issue). For our purposes, a selected indicator species (or species assemblage) should be sensitive to changes in deer browse pressure due to differences in deer populations or changes in management efforts (fencing, hunting, culling, etc.). Identification of indicator species that can be assessed regionally would also facilitate tracking long-term ecosystem recovery according to differences in the way deer populations are managed. We selected red oak (*Quercus rubra*) as our initial species of choice. The species is widespread in eastern North America, a major source of food with importance for wildlife (McShea *et al.* 2007; Tallamy and Shropshire 2009), an important timber species, of intermediate palatability (Averill *et al.* 2016) and easy to collect and propagate. In addition, red oaks are reported to show widespread regional regeneration failures due to excessive ungulate browse in eastern North America (Long *et al.* 2007; Abrams and Johnson 2012; Long *et al.* 2012; Thomas-Van Gundy *et al.* 2014; Leonardsson *et al.* 2015; Schwartz and Demchik 2015). But when deer numbers are kept lower, for example on tribal lands, red oaks flourish (Reo and Karl 2010).

We field-tested our indicator approach to assess importance of deer browse using planted and individually marked *Q. rubra* within a multiple stressor investigative framework (Fisichelli *et al.* 2013; Dávalos *et al.* 2015*a*, *b*). We evaluated the following hypotheses to assess utility and reliability of this method:

Hypothesis 1: Deer browse of *Quercus rubra* seedlings greatly exceeds impact of other stressors (particularly rodents, non-native earthworms and non-native plants).

Hypothesis 2: When protected from deer herbivory, *Quercus rubra* seedlings show positive growth rates under a full forest canopy regardless of presence of rodents, non-native plants or non-native earthworms.

## Methods

### Study location

We conducted our study at 12 forested sites located 1–8 km apart within US Army Garrison West Point, New York, USA. West Point is situated within the Hudson Highlands Province on the west bank of the Hudson River, ~80 km north of New York City, characterized by rugged hilly terrain with rocky outcrops and frequently thin soils. We were particularly interested to assess oak seedling survival in response to multiple stressors, including white-tailed deer, non-native earthworms and non-native invasive plants. At each location, we established two 30 m × 30 m paired plots situated 5–50 m apart from each other. One of the plots was randomly assigned to be fenced in July 2008 (Trident extruded deer fence, 2.3 m high, www.deerBusters.com, MD, USA). Sites differed in land-use history, aspect, soil and plant species composition, but within sites, paired plots had similar overstory and understory vegetation, and slope. Mature forest overstory consisted largely of oak (*Q. rubra* and *Q. prinus*) and/or sugar maple (*Acer saccharum*) but mostly lacked regenerating seedlings or saplings. We purposefully selected sites to include understories dominated by native plants (six sites) and sites where the understory vegetation was dominated by one of three non-native plants (*Alliaria petiolata*, *Berberis thunbergii* and *Microstegium vimineum*: two sites each). Sites also varied in invasive earthworm density and biomass but we had no *a priori* information on their abundance or species diversity. Similarly, we had no *a priori* information about the timing of earthworm invasions or local deer densities, but recreational hunting occurs at all our study locations.

### Red oak seedlings

We annually collected fallen red oak (*Q. rubra*) acorns in September and October from local sources at West Point. We floated acorns to assess viability, discarded floaters and stored acorns in gauze bags buried in moist sand in a walk-in environmental room (NORLAKE, Hudson, WI, USA) at 4 °C in the dark. In February/March of each year, we took acorns out of cold storage and planted them in individual SC7U Ray-Leach Cone-tainers (3.8 cm diameter × 14 cm deep; Stuewe and Sons, Tanget, OR, USA) using commercial potting soil (Fafard Canadian growing mix No. 1-P, Agawam, MA, USA). We arranged Cone-tainers in trays (98 per tray) placed in a heated greenhouse (20–25 °C daytime, 10 °C at night) under natural photoperiod. In late April or early May of each year, when seedlings had 2–4 leaves, we transferred trays outside to harden seedlings before transplanting into the field. We placed trays on elevated metal greenhouse benches with legs standing in buckets filled with soapy water, or on pallets above small artificial ponds, to prevent earthworm colonization. We protected seedlings against deer or rodent herbivory in walk-in field cages (Lumite® screening, shade 15 %, porosity 1629CFM; Synthetic Industries, Gainesville, GA, USA).

For planting at each site, we selected 40 seedlings with 3–8 leaves usually 8–15 cm tall. We watered all seedlings to saturation to create good starting conditions. At each site, we planted seedlings ≥3 m apart using multiple transects. We avoided planting seedlings next to live large trees or in windfalls, on very steep slopes or among large boulders that could function as refuges limiting physical access by deer. We used a handheld drill with a 5 cm diameter, 30 cm long, masonry drill bit to create tapered planting holes (10–15 cm deep × 5–10 cm wide). Before planting, we removed the acorn to reduce attraction to rodents, then planted seedlings in the hole and firmly covered potting soil with local soil. We placed a numbered metal tag (Racetrack aluminum tags; Forestry Suppliers, Jackson, MS, USA) staked into the ground next to each seedling. Immediately after planting, we measured seedling height (cm) and recorded the number of leaves.

At each site, we planted two cohorts of 3-month-old individually labelled red oak seedlings in 2010 and again in 2011. We planted 20 individuals each year into each open and fenced plot (40 per site, *N* = 920 individuals) and revisited all sites at regular intervals to record deer browse, rodent or insect attack, and mortality or survival, and occasionally the number of leaves and height of each individual.

We revisited each planting location after 7–10 days to assess mortality due to transplant shock (we did not lose any seedlings to transplant shock), and recorded deer browse, rodent attack, other herbivory or other causes of mortality (usually insects or pathogens). Thereafter, we visited plants at monthly intervals (in 2010) until the end of the growing season (October), to record deer browse, incidence of rodent attack and other sources of mortality. We revisited each location the following spring to record over winter mortality, deer browse or other mammal or invertebrate attack in mid–late May after leaf out. We repeated the same procedure in 2011, although intervals between visits were larger.

To assess influence of the understory plant community on oak seedling establishment, survival and growth, we used vegetation data recorded in 10 1-m^2^ stratified random permanent quadrats in each open and fenced plot established in July 2008 (11 sites) or May 2009 (1 site) (see Nuzzo *et al.* 2017, this issue). We recorded plant species presence, and estimated per cent cover for each species <1 m tall in mid-May and late July from 2008 to 2012 in 17 cover categories (midpoints: 0.01, 0.2, 0.5, 1, 3, 5, 10, 20, 30, 40, 50, 60, 70, 80, 90, 98 and 100 %) within each quadrat. We measured ‘average’ height of vegetation at four locations within each quadrat and then created a mean vegetation height for each plot. Surrounding vegetation could either function as above-ground competition or function as camouflage and hence protection of oak seedlings (Underwood *et al.* 2014).

To assess earthworm populations, we established five random 0.25 m^2^ quadrats per plot per year and poured 3.79 L of mustard solution at 15 g L^−1^ per quadrat (Frontier Natural Products Co-op, Norway, IA, USA) in mid-July 2008–2011 (Lawrence and Bowers 2002). We preserved surfacing earthworms in 70 % ethanol, identified mature individuals to species and then obtained ethanol-dried weight. Sites varied in earthworm abundance and diversity and for our analyses on their impact on oak survival or deer browse and rodent attack rates (see statistical analyses section), we categorized sites into low (1.4 *±* 0.37 individuals and 0.88 ± 0.36 g per 0.25 m^2^; means ± 1 SE; four sites) and high (12.4 ± 1.78 individuals and 8.6 ± 1.67 g per 0.25 m^2^; means ± 1 SE; eight sites) earthworm density categories. Earthworm abundance across sites did not follow a continuous distribution, rather sites grouped into two well-defined categories (as defined above). We only recorded non-native earthworm species. Common genera included *Amynthas*, *Dendrobaena*, *Aporrectodea*, *Octolasion* and *Lumbricus*.

### Oak herbivores

White-tailed deer are medium-sized browsers native to much of North and South America and the most widely distributed ungulate on the North American continent (Halls 1984; McCabe and McCabe 1984). White-tailed deer are selective foragers but their diet can be extremely variable based on local resources, seasons and nutritional requirements. The typical signs of browse are frayed edges (in contrast to clear cuts, see below) that indicate that deer grasped foliage and removed sections of or entire leaves, but most often the entire upper portion of a stem. We did encounter seedlings that were entirely ripped out of their planting holes. We scored these as deer attack based on deer feeding mode and lack of discernable excavation attempts. This mostly happened within a few weeks after planting when seedlings were not yet firmly anchored in the surrounding soil.

In our New York forests, the most common rodents are American red squirrel (*Tamiasciurus hudsonicus*), eastern grey squirrel (*Sciurus carolinensis*), white-footed mouse (*Peromyscus leucopus*), eastern chipmunk (*Tamias striatus*) and woodland vole (*Microtus pinetorum*). While we are unable to visually discriminate among feeding marks left by these species, all rodent attack can easily be distinguished from deer browse by the 45° cut angle that remains discernable until stems or tissues decompose. Furthermore, oaks are attacked by numerous guilds of insects attacking all plant tissues (borers, defoliators, gall makers) and diseases, but these rarely kill seedlings outright.

### Statistical analyses

To test our first hypothesis that deer herbivory overrides importance of other stressors, we used Cox proportional hazard models implemented in the R statistical (R Core Team 2016) package ‘coxme’ (Therneau 2015) to test effects of fencing (open or fenced), earthworm density (high or low), understory vegetation origin (native or non-native), understory vegetation height per plot and understory vegetation per cent cover per plot on rates of deer browse and rodent attack as well as unknown mortality. The exponent of the estimated coefficient in the model indicates the factor by which risk of an event increases/decreases in the observed group relative to the reference group (Therneau 2015). We included site and plot within site as random factors to reflect our nested design. The test compares time (number of days) to an event (for example, deer browse) among groups. No information about oak browse rates after the study period is available; therefore, data are considered right-censored. In order to avoid making inferences beyond the study period, we ran separate analyses for oaks planted in 2010 and 2011 because we monitored oaks planted in 2010 for a longer time period than oaks planted in 2011. We fitted separate models for deer browse, rodent attack and unknown mortality. We observed deer browse on three oaks planted in fenced plots after a fence breach in 2011. These oaks were excluded from further analyses and we did not include fencing in models evaluating rates of deer browse.

In the presence of competing risks (that is, deer browse, rodent attack and unknown mortality), Cox regressions are not adequate because they treat competing factors as censored observations (Scrucca *et al.* 2010). We used competing risk analysis (package ‘cmprsk’) (Gray 2014) to evaluate the probability of an event (for example, deer browse) occurring in presence of competing factors (rodent attack or unknown mortality). We include results from Cox regression and competing risk regression (CR) because CR does not account for random effects.

To test our second hypothesis that oak growth (increase in height) is limited by deer herbivory but positive even in the presence of other stressors, we evaluated effects of fencing, earthworm density and vegetation origin on oak height with linear mixed models (LMMs, package lme4) (Bates *et al.* 2014). We included site and plot within site as random factors in the model and included initial oak height (measured immediately after planting) and planting year (2010 or 2011) as covariates. For LMM only, we estimated conditional (full model) and marginal (fixed effects only) *R*^2^ (Nakagawa and Schielzeth 2013). Although power analysis is usually conducted before data collection, we ran the analysis in order to evaluate sample size using the package ‘simr’ (Green and MacLeod 2016). The power to reject the null hypothesis with an effect size of 1 cm was 89.3 % (95 % CI: 87.2 %–91.1 %) indicating adequate sample size.

We used Akaike Information Criterion (AIC; Burnham and Anderson 2002) to evaluate explanatory power among competing models (for LMM, Cox proportional hazard models and competing risk analysis). We ranked candidate models according to ΔAICc (difference between model’s AICc and min AICc). Lower ΔAICc indicates higher support for a given model. We considered all models within 2 AICc to be similar.

## Results

### Individual effects of deer herbivory, rodent attack and unknown mortality

**Figure 1.**
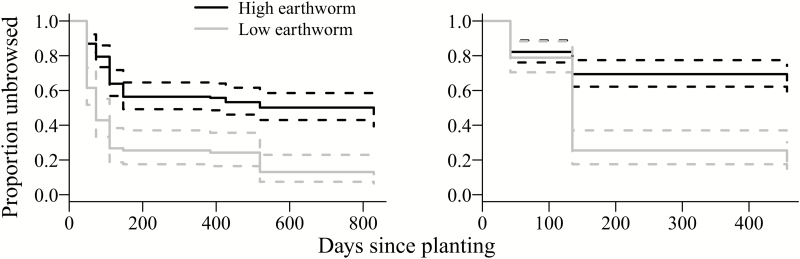
Proportion of oaks planted in 2010 (left) and 2011 (right) at 12 sites at West Point remaining unbrowsed by deer in open plots at sites with low (*N* = 4) and high (*N* = 8) earthworm density. We recorded no deer browse on oaks in fenced plots (see text for details). Solid lines represent expected values (dotted lines are 95 % CI) according to mixed effects Cox regression.

**Figure 2.**
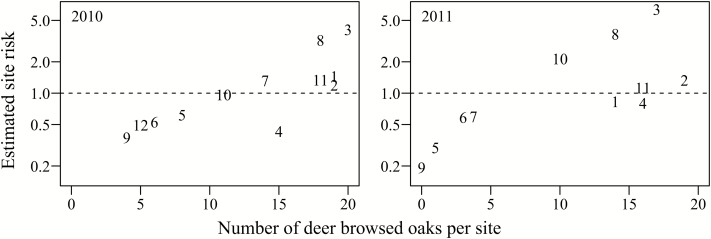
Risk of being browsed by deer in open plots at each study site according to mixed effects Cox regression. Twenty oaks were planted in each open plot at 12 sites at West Point in 2010 (left) and 2011 (right). Sites 1, 2, 4 and 11 have low earthworm density.

We monitored oaks planted in 2010 for 829 days after planting (last sampling date: 12 August 2012). We recorded rodent attack and other mortality factors on 51 oaks and deer browse on remaining oaks (157 out of 240 oaks planted in open plots) resulted in 83 % mean browse rate across sites. The median number of days an oak remained unbrowsed was 147 days (95 % CI: 110–519). In open plots, deer browse was significantly higher at sites with low earthworm density (89 % of oaks browsed) compared with sites with high earthworm density (54 % of oaks browsed; Fig. 1). At low earthworm sites, the probability of being browsed increased by 249 % (Table 1; **see Supporting Information—Table S1**). We found no significant effect of dominant herbaceous vegetation origin (native or non-native), vegetation height or vegetation cover **[see Supporting Information—Table S1]**. We recorded lowest risk of deer browse at four sites (sites 5, 6, 9, 12: Fig. 2), where only 4–8 individuals out of the 20 planted oaks were browsed. However, rodent attack and other mortality factors reduced the number of oaks available for deer consumption: at site 12 rodents attacked half of planted oaks (*N* = 10) and at site 5 six oaks in the open plot died for unknown reasons within 73 days of planting.

**Table 1.** Results for mixed effects Cox regression for deer browse on red oaks planted in open plots at 12 sites at West Point in 2010 and 2011. Only results for the best model are presented. Estimates and SEs are reported from the model fitted with restricted maximum likelihood. For model selection procedure, please **see Supporting Information—Table S1**. *Site 12 was excluded from analysis because all oaks in open plot were attacked by rodents within 42 days of planting.

**2010**				
**Fixed effects**	**Coef (SE**)	**Exp(coef**)	***z***	***P***
Earthworm (low)	1.25 (0.53)	3.49	2.34	0.02
**Random effects**	**SD**			
Site	0.83			
2011*				
**Fixed effects**	**Coef (SE**)	**Exp(coef**)	***z***	***P***
Earthworm (low)	1.57 (0.76)	4.78	2.06	0.04
**Random effects**	**SD**			
Site	1.15			

We monitored oaks in 2011 for 457 days after planting and we recorded deer browse on 113 individuals. We excluded site 12 from 2011 deer browse analyses because all oaks in the open plot were attacked by rodents within 42 days of planting. Similar to results from 2010 we found a significant effect of earthworm density and no significant effect of dominant herbaceous vegetation origin, vegetation height or vegetation cover (Table 1; **see Supporting Information—Table S1**; Fig. 1). At low earthworm sites, 81 % of oaks in unfenced plots were browsed compared with 30 % of oaks browsed at high earthworm sites.

For oaks planted in 2010, we recorded 49 rodent attacks over the 829 days period. Most attacks (*N* = 47) occurred within the first 73 days after planting. Rodent attack was present at all but one site (4), but was most prevalent at two sites (10 and 12). While at site 10 rodents attacked half of the oaks in the fenced plot (26 % and 203 % increased risk in open and fenced plot, respectively), at site 12 rodents attacked half of the oaks in the open plot (179 % and 33 % increased risk in open and fenced plot, respectively). Earthworm density, vegetation origin, vegetation height and vegetation cover had no effect on risk of rodent attack.

We monitored oaks planted in 2011 for 457 days and recorded 38 rodent attacks, most of them over the first 42 days. Oaks planted in the open plot at site 12 had a 1400 % increased risk of rodent attack and all 20 oaks planted in 2011 were attacked. At remaining sites rodents attacked one or two oaks per plot. Earthworm density, vegetation origin, vegetation height and vegetation cover had no effect on risk of rodent attack (best model included the random term only; **see Supporting Information—Table S2**).

**Figure 3.**
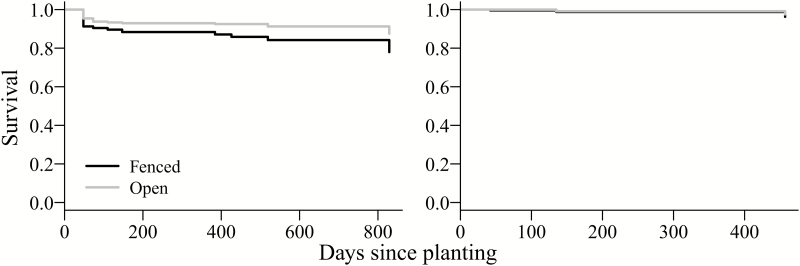
Survival of oaks (excluding individuals browsed by deer or attacked by rodents) planted in 2010 (left) and 2011 (right) in open and fenced plots at 12 sites at West Point. Mortality depicted here is due to unknown causes, including lack of survival over winter. Solid lines are expected values according to mixed effects Cox regression, CIs are omitted for clarity. Note that both lines on right-hand plot are overlapping.

In addition to deer browse and rodent attack, of the 480 oaks planted each year, 83 (2010) and 11 (2011) died of unknown causes (including winter mortality). Oaks planted in fenced plots in 2010 had a significant higher risk of death caused by unknown causes (Table 2; Fig. 3; 22 % recorded as dead) than oaks planted in the open plot (12 % recorded as dead). Fencing had no effect on incidence of unknown mortality affecting oaks planted in 2011. Incidence of unknown mortality was not affected by earthworm density, vegetation origin, vegetation height and vegetation cover for oaks planted in 2010 or 2011 **[see Supporting Information—Table S3]**.

**Table 2.** Results for mixed effects Cox regression for unknown mortality on red oaks planted in open and fenced plots at 12 sites at West Point in 2010 and 2011. Only results for the best model are presented. No study factor was significant for oaks planted in 2011. Estimates and SEs are reported from the model fitted with restricted maximum likelihood. For model selection procedure, please **see Supporting Information—Table S1**.

**2010**				
**Factor**	**Coef (SE**)	**Exp(coef**)	***z***	***P***
Fencing (open)	−0.72 (0.30)	0.49	−2.4	0.017
**Random effects**	**SD**			
Site/plot	0.41			
Site	0.84			

### Deer effects in the presence of other factors (rodent attack and unknown mortality)

**Figure 4.**
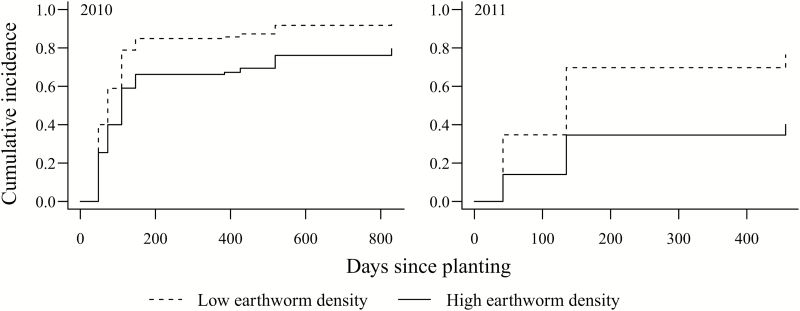
Cumulative incidence curve (accounting for rodent attack and mortality due to unknown causes) of the proportion of oaks browsed by deer over time (oaks planted in 2010, left panel; oaks planted in 2011, right panel) at high and low earthworm density sites (*N* = 12) at West Point.

After accounting for competing factors (rodent attack and mortality due to unknown causes), oaks planted in open plots, either in 2010 or 2011, had a higher risk of being browsed by deer at low earthworm density sites than at high earthworm density sites (Fig. 4). At low earthworm sites, the risk of being browsed increased by 63 % and 69 % in 2010 and 2011, respectively (Table 3; **see Supporting Information—Table S4**). Mean vegetation height in July (averaged across 20 quadrats per site) did not differ significantly (*F*_1,10_ = 0.002, *P* > 0.05) between sites with low (34.5 ± 15.3 cm; mean ± 1 SE) or high (33.5 ± 7.6 cm; mean ± 1 SE) earthworm density. However, overall, the risk of being browsed by deer significantly but slightly decreased (by 1 %) as a function of average vegetation height in either year (Table 3; **see Supporting Information—Table S4**). Vegetation origin and vegetation cover were not significant **[see Supporting Information—Tables S3 and S4]**.

**Table 3.** Results of cumulative risk analysis for oaks planted in open and fenced plots at 12 sites at West Point in 2010 and 2011. Only results for the best model are presented. For model selection procedure, please **see Supporting Information—Table S1.**

**2010**				
**Fixed effects**	**Coef (SE**)	**Exp(coef**)	***z***	***P***
Earthworm (low)	−0.99 (0.15)	0.37	−6.79	<0.001
Vegetation height	−0.01 (0.003)	0.99	−2.65	0.008
**2011**				
**Fixed effects**	**Coef (SE**)	**Exp(coef**)	***z***	***P***
Earthworm (low)	−1.17 (0.16)	0.31	−7.19	<0.001
Vegetation height	−0.01 (0.004)	0.99	−2.55	0.01

### Oak growth

**Figure 5.**
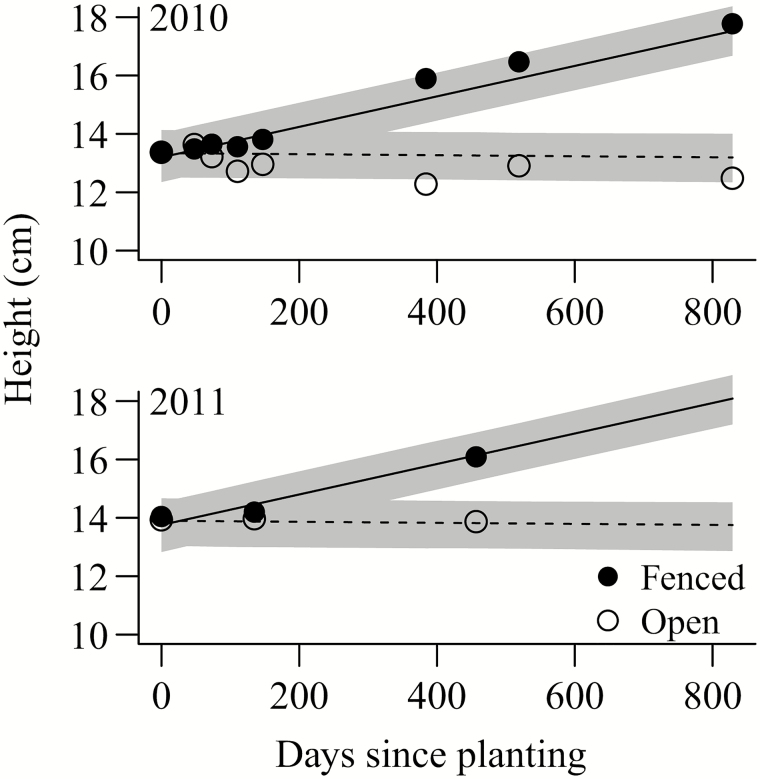
Height (cm) of oaks planted in 2010 (top) and 2011 (bottom) in open and fenced plots at 12 sites at West Point. We followed oaks planted in 2010 for 829 days, and those planted in 2011 for 457 days. Points represent mean height per plot at each sampling date, lines and shaded areas depict predictions and 95 % CI from linear mixed model. For model results, please see Table 4.

Oaks planted in 2010 were slightly but significantly shorter at planting (13.85 ± 0.20 cm; mean ± 1 SE) than oaks planted in 2011 (14.38 ± 0.13; mean ± 1 SE; *t*_760.9_ = −4.5, *P* < 0.001). In both years, oak height at planting was similar between open and fenced plots, but over time oaks in fenced plots grew significantly taller than oaks in open plots (Fig. 5; Table 4; significant interaction between time and fencing); conditional *R*^2^ [full model] = 0.68; marginal *R*^2^ [fixed factors] = 0.08). According to *R*^2^ results, most of the explained variation was contained in random factors. Oak height was not significantly affected by earthworm density or understory vegetation origin **[see Supporting Information—Table S5]**.

**Table 4.** Results of linear mixed model to evaluate effects of day since planting, planting year, fencing, earthworm density and vegetation origin on height (cm) of oaks planted in open and fenced plots at 12 sites at West Point in 2010 and 2011. Only results for the best model are presented. Estimates and SEs are reported from the model fitted with restricted maximum likelihood. For model selection procedure, please **see Supporting Information—Table S1.** *Chi-squared statistics and *P*-values are from likelihood ratio tests with each parameter removed from the maximum likelihood-based model, with all other parameters retained. It was not possible to test the significance of all terms because of higher-order interactions.

**Factor**	**Est**	**SE**	***t***	P*
Intercept	12.64	0.56	22.70	
Day	0.005	0.0001	27.22	
Fencing (open)	0.14	0.24	0.61	
Year planted	0.55	0.18	3.13	0.002
Day × fencing	−0.005	0.0003	−18.63	<0.001
**Random effects**	**SD**			
Site/plot/oak	6.16			
Site/plot	0.12			
Site	2.55			

## Discussion

Our experimental design and analyses allowed us to assess *Q. rubra* deer browse severity while simultaneously accounting for co-occurring factors, including stressors such as non-native earthworms and non-native plants. We thus accounted for other stressors that are frequently implicated as threats to native species (Simberloff *et al.* 2013; Craven *et al.* 2017) while isolating the magnitude of deer browse effects. We confirmed our first hypothesis and reports by others (Abrams and Orwig 1996; Abrams 2003; MacDougall *et al.* 2010; Thomas-Van Gundy *et al.* 2014) that deer browse is the overwhelming threat to recruiting *Q. rubra* seedlings, and that rodents, insects, winter mortality and introduced non-native plants and earthworms have a minor role in reducing annual seedling cohort survival. To the best of our knowledge, this is the first time that high deer browse has been confirmed as a severe threat to *Q. rubra* demography, while simultaneously accounting for multiple other threats. The importance of this finding cannot be overemphasized, particularly as recreational hunting occurred at all of our study locations, although hunting pressure varied by location and site accessibility. High deer browse pressure is the main factor that prevents future stand replacement of an important ecological and economic native species.

Our approach measures only one particular life stage in oak demography, the establishment of germinated seedlings over the first few years of a species that may live for centuries. But it is exactly this stage that is most vulnerable to deer herbivory and competition with invasive plants species and is missing from many forests, even where overstories are dominated by oaks (Thomas-Van Gundy *et al.* 2014), as at our study locations. And the browse intensity on these small seedlings is likely underestimated by most foresters or landowners as most small seedlings are eaten before they can be censused. At our study locations, we rarely encountered recruiting oak seedlings despite the high frequency of mature oaks in the overstory (unpublished data) and regular masting events. Future replacement of large canopy oaks depends on seedlings and saplings that persist in the understory for decades while awaiting an opportunity to grow into the canopy (Thomas-Van Gundy *et al.* 2014). Decreased light levels in many regenerating forests are frequently cited as the dominant successional force that favours shade and browse-tolerant species (Lorimer 1984; Nuttle *et al.* 2011; Thomas-Van Gundy *et al.* 2014; Leonardsson *et al.* 2015; Schwartz and Demchik 2015). However, our results also show clearly that seedling oaks are growing even in shaded conditions under a full forest canopy, albeit slowly. At growth rates of 1–2 cm per year, seedlings need to survive for extended periods in forests, unless light conditions become favourable, to reach a height where they are less vulnerable to deer browse. At present, and without an existing demographic model for *Q. rubra*, it is difficult to establish an annual maximum threshold of seedling browse rates that could reliably sustain populations. Further work to develop more sophisticated demographic models, such as those that are available for white trillium (*Trillium grandiflorum*) or American ginseng (*Panax quinquefolius*) (McGraw and Furedi 2005; Knight *et al.* 2009*a*), is needed to establish thresholds that are informed by threats to red oaks and other species of economic and conservation concerns.

Introduced invasive plants are often considered major threats to ecosystems around the world (Mack *et al.* 2000; Simberloff *et al.* 2013), although our views and the evidence of introduced species as drivers of ecosystem change are evolving (MacDougall and Turkington 2005; Davis *et al.* 2011). This is a particularly important research endeavour given the prevalence of multiple stressors that simultaneously may affect native species growth and survival (Fisichelli *et al.* 2013; Dávalos *et al.* 2015*a*, *b*). We were able to assess the influence of invasive earthworms and three major forest plant invaders on *Q. rubra* establishment and growth. We found no direct link to oak seedling survival or performance, thus further supporting our first hypothesis. However, indirectly, the linkage of high deer populations to increased abundance of invasive plants and earthworms that may threaten other plant species of conservation concern becomes increasingly clear (Eschtruth and Battles 2008; Eschtruth and Battles 2009; Knight *et al.* 2009*b*; Heckel *et al.* 2010; Kalisz *et al.* 2014; Dávalos *et al.* 2015*a*, *b*). At one site where we planted *Q. rubra* into dense *M. vimineum*, seedlings in the open plot suffered catastrophic mortality due to rodents attacking all seedlings (site 12 in 2011). We hypothesize that the dense cover of *M. vimineum* locally elevated either rodent activity or the rodent population.

Similarly, while earthworms did not directly affect growth or survival of protected seedlings, at low earthworm sites *Q. rubra* seedlings had an elevated risk of being browsed, even when accounting for other mortality factors (Figs. 1 and 4), an indirect pathway that we did not account for in our first hypothesis. We hypothesize that this elevated browse risk is a function of site conditions, with earthworms colonizing richer sites with increased non-native vegetation cover and higher soil pH (Dávalos *et al.* 2015*c*). We are currently unable to provide further explanations or mechanisms for this finding, but the effect is clearly ecologically relevant. Whether deer favour sites or site conditions that repel earthworms, or whether high earthworm populations provide oak seedlings protection against deer browse through increased ability to produce chemical deterrents, for example, remain open questions for future investigations. Importantly, our oak sentinel approach using standardized assessments across multiple sites that differ in site conditions and deer browse pressure captured this effect, demonstrating the importance of a standardized approach that does not rely on pre-existing vegetation.

Our data provide clear evidence that efforts to reduce deer abundance and impact are needed to safeguard the more palatable and/or less browse-resistant species that continue to suffer at present deer densities (Knapp and Wiegand 2014). All our sites at West Point allow access by recreational hunters, but that did not prevent high deer browse rates of oak seedlings. While recreational hunting can reduce deer populations (Stewart *et al.* 2013; Williams *et al.* 2013) and resulting browse damage in the USA (Jenkins *et al.* 2014, 2015) and Europe (Hothorn and Müller 2010), the improvements are measurable but not necessarily biologically relevant for the most browse-sensitive species. Negative effects on forest ecosystems and their biota are evident at much lower deer abundance than currently exists over much of the northeastern USA (Horsley *et al.* 2003) and these conditions of changed forest trajectories will be long-term (Nuttle *et al.* 2011). For example, initiation of a hunting programme in several parks in Indiana reduced deer browse impacts and increased cover of native and reduced cover of non-native species, but browse-sensitive herbaceous species, such as *Trillium* spp., were lost (Jenkins *et al.* 2014). Similarly, while *Quercus* spp. were common overstory species in these parks, they failed to recruit into the seedling or sapling class even after a decade of sustained hunting pressure (Jenkins *et al.* 2015).

The question that puzzles most ecologists and conservationists is that in the face of overwhelming evidence (Martin *et al.* 2011) wildlife managers in North America have not responded strongly enough to the threat posed by abundant deer. This is due in great part to the fact that hunters have an outsized influence on wildlife management decisions. Most hunters do not believe, or are not aware of, the extent of impacts deer have on forest ecosystems (Diefenbach and Palmer 1997; Diefenbach *et al.* 1997). Management agencies rely on recreational hunting as a management tool (The Wildlife Society 2016) and this enjoys widespread public support particularly for meat (versus trophy) hunting (Decker *et al.* 2015). However, recreational hunting does not effectively reduce populations to ecological carrying capacity, i.e. to where deer no longer represent threats to other biota (Horsley *et al.* 2003; Latham *et al.* 2009; Williams *et al.* 2013; Boulanger *et al.* 2014; Dávalos *et al.* 2015*a*). In addition, animal welfare and animal rights organizations, for very different reasons, similarly reject the overwhelming evidence (HSUS 2016) leading to increasingly bitter fights over appropriate management goals and techniques (Frye 2006; Cambronne 2012; Sterba 2012; The Wildlife Society 2016).

It is evident that current wildlife management has failed to provide the protection needed by species that are vulnerable to ungulate browse, and this is true not only for North America and white-tailed deer but also for a number of ungulates in Europe, New Zealand and Australia (Ammer *et al.* 2010; Putman *et al.* 2011; Tanentzap *et al.* 2011; Wright *et al.* 2012; Howland *et al.* 2014; Grünwald *et al.* 2016). The acceptance of regulated commercial hunting to encourage further deer population reductions is receiving increased consideration (Vercauteren *et al.* 2011) although in North America prohibition of unregulated commercial hunting is a cornerstone of the North American Model of Wildlife Conservation (Geist *et al.* 2001). In contrast, the ability to sell game meats to reward increased hunter efforts is widely available in Europe (Ljung *et al.* 2012).

Our oak indicator species planting is one step towards developing a standardized approach to measure deer browse impacts (as opposed to deer abundance). We were particularly interested to test an approach that could be implemented by local landowners or in a citizen science framework (Cooper *et al.* 2007; Silvertown 2009; Dickinson *et al.* 2010) to allow widespread application. Furthermore, impact perception and knowledge is important for social acceptance of deer management (Lischka *et al.* 2008), and beliefs about ecological impact predict attitudes towards deer management (Johnson and Horowitz 2014). Active involvement and development of local expertise and local evidence will increase ecological literacy and acceptance of findings. Instead of relying on expert opinion and data from distant places, personal experiences can transform our interactions and relationships with the environment (Novacek 2008) and provide opportunities for enhanced understanding. Our oak sentinel approach is encouraging exactly this engagement and can empower individuals to make choices based on sound evidence. It measures local effects and, if desired, negative effects can be locally remedied. However, it also requires the willingness by state wildlife agencies to empower local landowners and municipalities to pursue approaches that they deem appropriate.

We have shown that we can measure differences in local deer browse pressure using *Q. rubra* seedlings (Fig. 2) and we are currently experimenting with additional herbaceous species (B. Blossey, V. Nuzzo, A. Dávalos, unpublished data) to supplement the evidence collected from oaks and woody vegetation. Similar efforts using existing vegetation (Chevrier *et al.* 2012) or plantings and fencing to assess deer browse pressure have been made for other oak species (Gonzales and Arcese 2008; MacDougall *et al.* 2010; Long *et al.* 2012) and Canada yew (*Taxus canadensis*), a highly preferred species that has suffered precipitous declines due to deer browse (Holmes *et al.* 2009). There is additional work to be done to test this or similar approaches in a scaled-up version to address landscape level or regional threat assessments. And it is clear that additional species, for example more browse-susceptible and herbaceous plants, will need to become part of an assessment framework in addition to red oaks. The list of proposed woody and herbaceous species to measure deer impacts is substantial (Anderson 1994; Balgooyen and Waller 1995; Webster and Parker 2000; Williams *et al.* 2000; Fletcher *et al.* 2001; Webster *et al.* 2001; Kraft *et al.* 2004; Kirschbaum and Anacker 2005; Krueger and Peterson 2006; Pierson and DeCalesta 2015; Royo *et al.* 2016), yet all rely on the continued availability of these species in the landscape. Planting individuals of similar size and age offers an intriguing alternative where these species no longer exist and can complement restoration efforts. In some of our ongoing work, we have documented extremely favourable oak seedling survival in locales where *T. grandiflorum* are browsed at unsustainable levels for positive population growth rates (A. Dávalos, B. Blossey, V. Nuzzo, unpublished data).

We are not aware of any system in North America or elsewhere where routine deer damage assessment of important conservation targets is being conducted. While long-term vegetation monitoring plots exist at many locations, they cannot account specifically for deer effects because other stressors, such as earthworms, rodents, etc., remain unaccounted for (although, as we show here, these other stressors have low explanatory power in our study forests). A review of ungulate browse assessment schemes in Europe (Reimoser and Putman 2011) shows that while some regional damage assessments are done for agriculture and forestry (Ammer *et al.* 2010; Hothorn and Müller 2010; Grünwald *et al.* 2016), there are no data to assess the realized impacts of deer on non-commercial plants and associated biota (Putman *et al.* 2011). Without evidence and full accountability the quagmire of deer management will continue to persist in North America and Europe (Perea *et al.* 2014). This is an unacceptable status quo and we hope that implementation of an indicator species approach, such as our oak sentinels, will help develop a more effective stewardship approach—one based on accountability, evidence and public trust principles (Hare and Blossey 2014).

## Conclusions

Foresters, ecologists, conservationists, animal rights groups, suburban communities, hunters and local, state and federal land management agencies argue about appropriate population targets for *O. virginianus*. Unfortunately, local evidence documenting the extent of impacts on individual plants, animals, ecosystems and food webs is largely absent, yet the impacts ungulates have on ecosystems and local biota should guide their management. Evidence is accumulating that impacts are not only related to current deer densities but also can be a function of deer legacy effects, differences in plant community composition and soil fertility or local landscape context. Our standardized sentinel approach to assess impact of deer browse, while incorporating the importance of rodent attack, invasive earthworms and invasive plants on survival and growth of 3-month-old red oak (*Q. rubra*) individuals, found strong year and site-specific effects with catastrophic browse of unprotected individuals (70–90 % of individuals over a 2.5-year observational period), far exceeding the importance of any other factor. Oaks planted at low earthworm density sites were at significantly higher risk of being browsed by deer compared with oaks at high earthworm density sites but there was no detectable negative effect of invasive plant species. Surviving oaks grew (~2 cm per year) under full forest canopy cover, but only when fenced. We recommend assessing deer impacts on plants using marked individuals and their performance and hope that similar protocols can be developed to assess impacts on other biota. The strength of this approach is that typical problems associated with multiple stressor impacts can be avoided, areas devoid of vegetation but under heavy deer browse pressure can still be assessed and the method can be implemented by non-specialists. Furthermore, this data collection can allow development of sophisticated demographic models and ecological forecasting if used for other species.

## Sources of Funding

Funding was provided by the Strategic Environmental Research and Development Program (SERDP) of the U.S. Department of Defense (grant RC-1542 to B.B.).

## Contributions by the Authors

B.B. and V.N. conceived the West Point study design; B.B. developed and implemented the oak sentinel idea. B.B. collected data on oaks; V.N. collected plant data; and A.D. and V.N. collected earthworm data. A.D. conducted all analyses and prepared the figures. B.B. led the writing and all authors participated in writing the manuscript.

## Conflicts of Interest

None declared.

## Supplementary Material

Supporting Information
