# Supplementary material for: An indicator approach to capture impacts of white-tailed deer and other ungulates in the presence of multiple associated stressors
Source: AoB Plants. 2017 Jul 22;9(5):plx034. doi: 10.1093/aobpla/plx034 (PMC5585856; doi:10.1093/aobpla/plx034)
Supplement: Supporting Information [file plx034_Supporting_Information.docx]

**SUPPORTING INFORMATION**

TABLE S1. Results of Mixed Effects Cox Regression for deer herbivory on red oaks planted in open and fenced plots at 12 sites at West Point in 2010 and 2011. We excluded site 12 from 2011 analysis because all oaks in the open plot were attacked by rodents within 42 d of planting. All models included site as random effect. Models are ordered according to lowest AIC value ([Burnham and Anderson 2002](#_ENREF_1)). dAIC is the difference between the AICc of a particular model compared to the lowest AICc observed. The Akaike weight is calculated as the model likelihood normalized by the sum of all model likelihoods; values close to 1.0 indicate greater confidence in the selection of a model. Only models within 2 AICc are considered.

| **Year planted** | **Factors** | **df** | **LogLik** | **AIC_c_** | **ΔAIC_c_** | **Weight** |
| --- | --- | --- | --- | --- | --- | --- |
| 2010 | Earthworms | 9 | -732.24 | 1485.01 | 0.00 | 0.19 |
|  | Cover, earthworms, site invasion | 9 | -732.80 | 1486.16 | 1.15 | 0.11 |
|  | Cover, earthworms | 9 | -732.84 | 1486.26 | 1.25 | 0.10 |
|  | Earthworms, site invasion | 9 | -732.84 | 1486.45 | 1.44 | 0.09 |
|  | Earthworms, vegetation height, site invasion | 9 | -732.88 | 1486.49 | 1.48 | 0.09 |
|  | Cover, earthworms, vegetation height, site invasion | 10 | -732.82 | 1486.62 | 1.61 | 0.08 |
|  | Cover | 10 | -732.88 | 1486.92 | 1.91 | 0.07 |
|  | Null | 10 | -732.87 | 1487.00 | 1.99 | 0.07 |
|  | Site invasion | 10 | -732.88 | 1487.13 | 2.12 | 0.07 |
|  | Vegetation height | 10 | -732.88 | 1487.14 | 2.13 | 0.07 |
|  | Cover, vegetation height | 10 | -732.88 | 1487.19 | 2.19 | 0.06 |
|  |  |  |  |  |  |  |
| **Year planted** | **Factors** | **df** | **LogLik** | **AIC_c_** | **ΔAIC_c_** | **Weight** |
| 2011 | Earthworms | 8 | -520.13 | 1058.72 | 0.00 | 0.17 |
|  | Earthworms, vegetation height | 8 | -520.34 | 1059.31 | 0.59 | 0.13 |
|  | Cover | 9 | -520.10 | 1059.44 | 0.72 | 0.12 |
|  | Vegetation height | 9 | -520.11 | 1059.50 | 0.78 | 0.12 |
|  | Cover, vegetation height | 9 | -520.09 | 1059.58 | 0.86 | 0.11 |
|  | Cover, earthworms, vegetation height | 8 | -520.56 | 1059.78 | 1.06 | 0.10 |
|  | Cover, earthworms, vegetation height, site invasion | 9 | -520.62 | 1060.20 | 1.48 | 0.08 |
|  | Null | 9 | -520.83 | 1060.75 | 2.03 | 0.06 |

TABLE S2. Results of Mixed Effects Cox Regression for rodent attacks on red oaks planted in open and fenced plots at 12 sites at West Point in 2010 and 2011. All models included site and plot within site as random effects. Models are ordered according to lowest AIC value ([Burnham and Anderson 2002](#_ENREF_1)). dAIC is the difference between the AICc of a particular model compared to the lowest AICc observed. The Akaike weight is calculated as the model likelihood normalized by the sum of all model likelihoods; values close to 1.0 indicate greater confidence in the selection of a model.

| **Year planted** | **Factors** | **df** | **LogLik** | **AIC_c_** | **ΔAIC_c_** | **Weight** |
| --- | --- | --- | --- | --- | --- | --- |
| **2010** | Cover, earthworms, vegetation height | 12 | -276.89 | 579.45 | 0.00 | 0.14 |
|  | Cover | 12 | -276.72 | 579.55 | 0.10 | 0.13 |
|  | Earthworms | 12 | -276.81 | 579.72 | 0.27 | 0.12 |
|  | Cover, earthworms | 12 | -276.76 | 579.79 | 0.34 | 0.12 |
|  | Null | 12 | -276.80 | 579.99 | 0.54 | 0.11 |
|  | Site invasion | 13 | -276.66 | 580.24 | 0.79 | 0.09 |
|  | Cover, earthworms, vegetation height, fencing | 12 | -276.89 | 580.49 | 1.04 | 0.08 |
|  | Earthworms, vegetation height | 12 | -276.91 | 580.58 | 1.13 | 0.08 |
|  | Vegetation height | 13 | -276.85 | 580.79 | 1.34 | 0.07 |
|  | Cover, vegetation height | 13 | -276.89 | 581.31 | 1.86 | 0.05 |
|  |  |  |  |  |  |  |
| **Year planted** | **Factors** | **df** | **LogLik** | **AIC_c_** | **ΔAIC_c_** | **Weight** |
| 2011 | Cover, vegetation height | 9 | -176.89 | 372.49 | 0.00 | 0.24 |
|  | Cover, earthworms, vegetation height | 9 | -177.00 | 372.80 | 0.31 | 0.21 |
|  | Null | 11 | -174.43 | 373.00 | 0.51 | 0.19 |
|  | Cover, earthworms, fencing, vegetation height | 9 | -177.33 | 373.75 | 1.25 | 0.13 |
|  | Cover, earthworms, fencing, vegetation height, site invasion | 9 | -177.62 | 374.58 | 2.09 | 0.09 |
|  | Cover | 11 | -175.66 | 374.87 | 2.38 | 0.07 |
|  | Cover, earthworms | 12 | -175.56 | 375.82 | 3.33 | 0.05 |
|  | Site invasion | 13 | -175.18 | 379.24 | 6.75 | 0.01 |
|  | Earthworms | 14 | -174.77 | 379.49 | 7.00 | 0.01 |
|  | Earthworms, vegetation height | 14 | -174.74 | 379.67 | 7.18 | 0.01 |
|  | Vegetation height | 13 | -176.10 | 380.14 | 7.64 | 0.01 |

TABLE S3. Results of Mixed Effects Cox Regression for unknown mortality on red oaks planted in open and fenced plots at 12 sites at West Point in 2010 and 2011. All models included site and plot within site as random effects. Models are ordered according to lowest AIC value ([Burnham and Anderson 2002](#_ENREF_1)). dAIC is the difference between the AICc of a particular model compared to the lowest AICc observed. The Akaike weight is calculated as the model likelihood normalized by the sum of all model likelihoods; values close to 1.0 indicate greater confidence in the selection of a model.

| **Year planted** | **Factors** | **df** | **LogLik** | **AIC_c_** | **ΔAIC_c_** | **Weight** |
| --- | --- | --- | --- | --- | --- | --- |
| **2010** | Cover, earthworms, fencing | 11 | -472.48 | 967.53 | 0.00 | 0.16 |
|  | Cover, fencing | 11 | -472.33 | 967.56 | 0.03 | 0.16 |
|  | Fencing | 13 | -470.42 | 967.81 | 0.27 | 0.14 |
|  | Cover, fencing, site invasion | 11 | -472.56 | 968.16 | 0.63 | 0.12 |
|  | Cover, earthworms, fencing, vegetation height | 11 | -472.56 | 968.24 | 0.70 | 0.11 |
|  | Cover, earthworms, fencing, vegetation height, site invasion | 11 | -472.64 | 968.95 | 1.41 | 0.08 |
|  | Earthworms | 13 | -471.36 | 969.51 | 1.98 | 0.06 |
|  | Cover | 13 | -472.66 | 972.58 | 5.05 | 0.01 |
|  | Null | 15 | -471.55 | 974.34 | 6.80 | 0.01 |
|  |  |  |  |  |  |  |
| **Year planted** | **Factors** | **df** | **LogLik** | **AIC_c_** | **ΔAIC_c_** | **Weight** |
| 2011 | Cover, vegetation height | 7 | -85.35 | 185.48 | 0.00 | 0.20 |
|  | Null | 6 | -86.66 | 186.17 | 0.69 | 0.14 |
|  | Cover, earthworms, fencing, vegetation height, site invasion | 7 | -85.55 | 186.60 | 1.12 | 0.12 |
|  | Cover | 6 | -86.43 | 186.82 | 1.34 | 0.10 |
|  | Earthworms, fencing | 7 | -86.20 | 187.02 | 1.54 | 0.09 |
|  | Fencing | 7 | -86.32 | 187.24 | 1.76 | 0.08 |
|  | Cover, earthworms, fencing | 8 | -85.50 | 187.34 | 1.86 | 0.08 |
|  | Cover, fencing | 7 | -85.98 | 187.70 | 2.22 | 0.07 |
|  | Cover, earthworms, fencing, site invasion | 8 | -85.65 | 188.48 | 3.00 | 0.05 |
|  |  |  |  |  |  |  |

TABLE S4. Results of Mixed Effects Cox Regression for unknown mortality on red oaks planted in open and fenced plots at 12 sites at West Point in 2010 and 2011. All models included site and plot within site as random effects. Models are ordered according to lowest AIC value ([Burnham and Anderson 2002](#_ENREF_1)). dAIC is the difference between the AICc of a particular model compared to the lowest AICc observed. The Akaike weight is calculated as the model likelihood normalized by the sum of all model likelihoods; values close to 1.0 indicate greater confidence in the selection of a model.

| **Year planted** | **Factors** | **df** | **LogLik** | **AIC_c_** | **ΔAIC_c_** | **Weight** |
| --- | --- | --- | --- | --- | --- | --- |
| **2010** | Earthworm, vegetation height | 2 | -784.59 | 1573.19 | 0 | 0.47 |
|  | Cover, earthworms, vegetation height, site invasion | 4 | -782.85 | 1573.7 | 0.51 | 0.37 |
|  | Earthworms, vegetation height, site invasion | 3 | -784.69 | 1575.37 | 2.19 | 0.16 |
|  | Earthworm | 1 | -790.91 | 1583.82 | 10.64 | 0 |
|  | Earthworm, site invasion | 2 | -790.62 | 1585.25 | 12.06 | 0 |
|  | Site invasion | 1 | -804.56 | 1611.13 | 37.94 | 0 |
|  | Vegetation height | 1 | -805.39 | 1612.78 | 39.59 | 0 |
|  | Null | 0 | -806.68 | 1613.36 | 40.17 | 0 |
|  |  |  |  |  |  |  |
| **Year planted** | **Factors** | **df** | **LogLik** | **AIC_c_** | **ΔAIC_c_** | **Weight** |
| 2011 | Earthworm, vegetation height | 2.00 | -581.96 | 1167.91 | 0.00 | 0.46 |
|  | Earthworms, vegetation height, site invasion | 3.00 | -581.90 | 1169.80 | 1.88 | 0.18 |
|  | Cover, earthworms, vegetation height, site invasion | 4.00 | -581.05 | 1170.10 | 2.19 | 0.15 |
|  | Earthworm, site invasion | 2.00 | -583.34 | 1170.68 | 2.77 | 0.12 |
|  | Earthworm | 1.00 | -584.61 | 1171.21 | 3.30 | 0.09 |
|  | Vegetation height | 1.00 | -595.21 | 1192.42 | 24.51 | 0.00 |
|  | Site invasion | 1.00 | -600.34 | 1202.69 | 34.78 | 0.00 |
|  | Null | 0.00 | -806.68 | 1613.36 | 445.44 | 0.00 |

TABLE S5. Results of Linear Mixed Model for Vegetation height of red oaks planted in open and fenced plots at 12 sites at West Point in 2010 and 2011. All models included site and plot within site as random effects. Models are ordered according to lowest AIC value ([Burnham and Anderson 2002](#_ENREF_1)). dAIC is the difference between the AICc of a particular model compared to the lowest AICc observed. The Akaike weight is calculated as the model likelihood normalized by the sum of all model likelihoods; values close to 1.0 indicate greater confidence in the selection of a model.

| **Factors** | **df** | **LogLik** | **AIC_c_** | **ΔAIC_c_** | **Weight** |
| --- | --- | --- | --- | --- | --- |
| Day, fencing, year planted, day*fencing | 9 | -10411.69 | 20841.42 | 0.00 | 1.00 |
| Day, fencing, year planted, site invasion, day*fencing | 10 | -10411.60 | 20843.25 | 1.83 | 0.40 |
| Day, fencing, earthworms, year planted, site invasion, day*fencing | 11 | -10411.05 | 20844.17 | 2.75 | 0.25 |
| Day, fencing, earthworms, year planted, site invasion, day*fencing | 12 | -10410.40 | 20844.87 | 3.44 | 0.18 |
| Day, fencing, earthworms, year planted, site invasion, day*fencing, fencing*site invasion, fencing*earthworm | 13 | -10410.21 | 20846.50 | 5.08 | 0.08 |
| Day(Q), fencing, earthworms, year planted, site invasion, day*fencing, fencing*site invasion, fencing*earthworm | 14 | -10409.78 | 20847.67 | 6.24 | 0.04 |
| Day, fencing, day*fencing | 8 | -10416.55 | 20849.12 | 7.70 | 0.02 |
| Fencing, year planted | 7 | -10748.86 | 21511.74 | 670.31 | 0.00 |

**LITERATURE CITED**

Burnham KP, Anderson DR. 2002. Model Selection and Multimodel Inference: A Practical Information-Theoretic Approach. Springer, New York, New York, USA.
